# Garner’s aldehyde as a versatile intermediate in the synthesis of enantiopure natural products

**DOI:** 10.3762/bjoc.9.300

**Published:** 2013-11-26

**Authors:** Mikko Passiniemi, Ari MP Koskinen

**Affiliations:** 1 Aalto-University, School of Chemical Technology, Department of Chemistry P.O. Box 16100 (Kemistintie 1), FI-00076 Aalto, Finland

**Keywords:** asymmetric synthesis, Garner’s aldehyde, natural product synthesis, L-serine

## Abstract

Since its introduction to the synthetic community in 1984, Garner’s aldehyde has gained substantial attention as a chiral intermediate for the synthesis of numerous amino alcohol derivatives. This review presents some of the most successful carbon chain elongation reactions, namely carbonyl alkylations and olefinations. The literature is reviewed with particular attention on understanding how to avoid the deleterious epimerization of the existing stereocenter in Garner’s aldehyde.

## Introduction

“*The universe is a dissymmetrical whole. I am inclined to think that life, as manifested to us, must be a function of the dissymmetry of the universe and of the consequences it produces. The universe is dissymmetrical; for, if the whole of the bodies which compose the solar system were placed before a glass moving with their individual movements, the image in the glass could not be superimposed on reality. Even the movement of solar life is dissymmetrical. A luminous ray never strikes in a straight line the leaf where vegetable life creates organic matter [...] Life is dominated by dissymmetrical actions. I can even foresee that all living species are primordially, in their structure, in their external forms, functions of cosmic dissymmetry.*“ [1]

- Louis Pasteur

**Figure 1:**
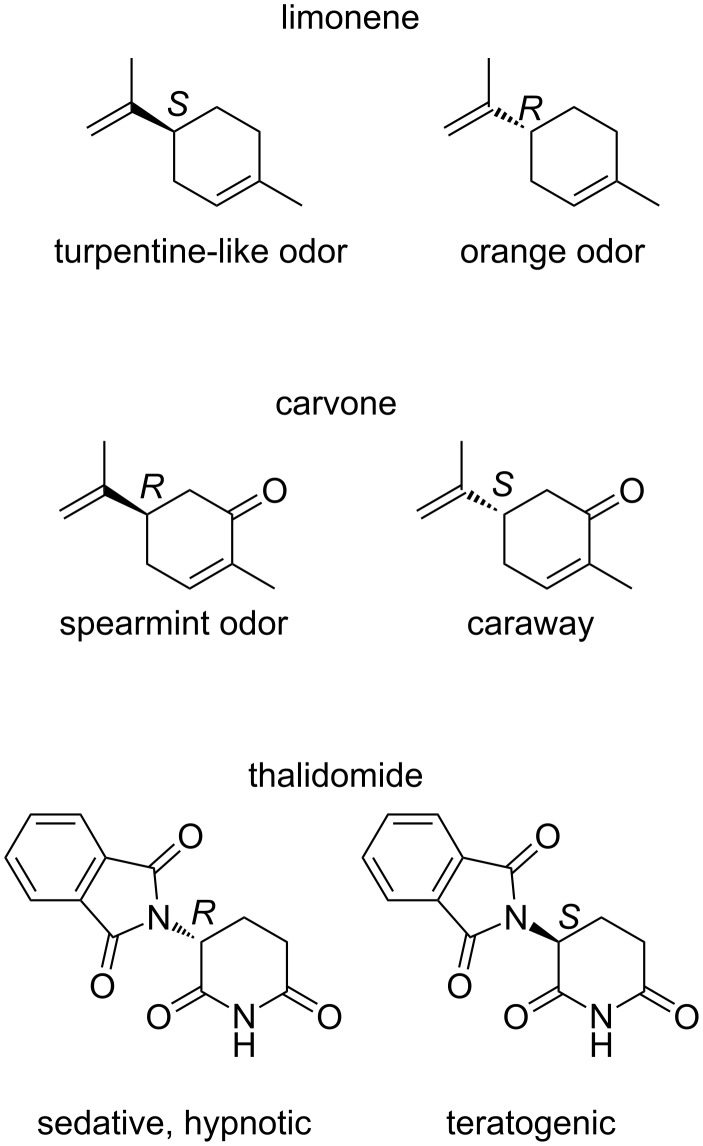
Structures of limonene, carvone and thalidomide.

These visionary words were written over 100 years ago by Louis Pasteur. Little did he know how great of a challenge underlies these words. Natural products, secondary metabolites produced by living organisms, have their own distinct structures. Some of them have the same chemical structure, but differ from each other only by being mirror images (e.g., (*R*)(+)/(*S*)(−)-limonene and (*S*)(+)/(*R*)(–)-carvone, Figure 1). This sounds like an insignificant difference, but in reality enantiomers can have a totally different, even contradictory, effect on living organisms. As an example, (*R*)-limonene smells of oranges, whereas the (*S*)-enantiomer has a turpentine-like (with a lemon note) odor. The difference in physiological effects of enantiomers is of utmost importance especially for the pharmaceutical industry, but increasingly also in agrochemicals [2–3] and even in materials sciences, as evidenced by the introduction of chiral organic light emitting diodes [4–5]. In certain cases, one enantiomer may be harmful. This was the case, for example, with the drug thalidomide (Figure 1), the (*R*)-enantiomer of which was sold to pregnant women as a sedative and antiemetic in the 1960s. The (*S*)-enantiomer turned out to be teratogenic.

The crucial role of chirality presents a great challenge for synthetic chemists. Asymmetric synthetic methods have emerged and after the development of ample analytical methods over the last few decades, asymmetric synthesis has seen an exponential growth. This has allowed us to tackle even more challenging targets like palytoxin [6–8], vinblastine [9–14], and paclitaxel [15–23]. The field is still far from being mature, and there remains a huge demand for more advanced methods for the introduction of chirality to substrates.

This review presents a general overview of the synthesis and use of Garner’s aldehyde in natural product synthesis. Particular attention will be paid on the preservation of chiral information in the addition reaction of nucleophiles to the aldehyde. Models are presented for understanding the factors affecting the stability of the stereocenters, as well as those affecting diastereoselectivity in the generation of the new stereocenter.

## Review

**Figure 2:**
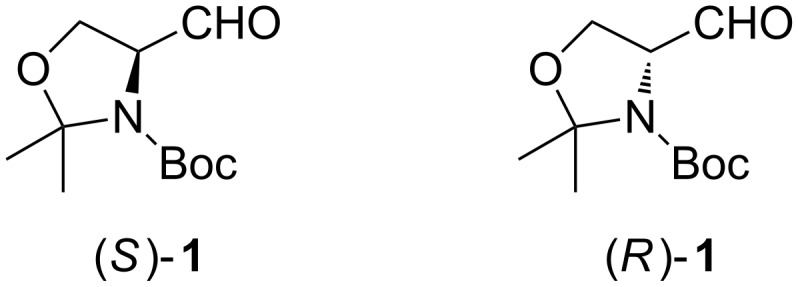
Structure of Garner’s aldehyde.

Philip Garner was the first to report a synthesis for 1,1-dimethylethyl 4-formyl-2,2-dimethyloxazolidine-3-carboxylate (**1**, Figure 2), today better known as Garner’s aldehyde [24–25]. This configurationally stable aldehyde has shown its power as a chiral building block in the synthesis of various natural products as well as their synthetic intermediates. It is one of the most cited chiral building blocks in recent times and has been used in over 600 publications.

### Synthesis of Garner’s aldehyde

Garner’s aldehyde (**1**) has been widely used as an intermediate in multistep synthesis. Thus the synthesis of **1** has to meet some essential requirements: 1) easy and large scale preparation and 2) configurational and chemical stability of all intermediates.

**Scheme 1:**
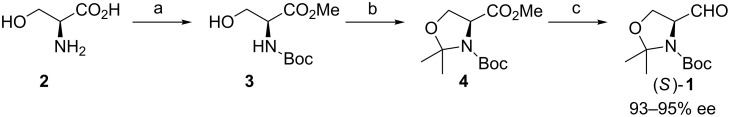
**(**a) i) Boc_2_O, 1.0 N NaOH (pH >10), dioxane, +5 °C → rt; ii) MeI, K_2_CO_3_, DMF, 0 °C → rt (86% over two steps); (b) Me_2_C(OMe)_2_, cat. *p*-TsOH, benzene, reflux (70–89%); (c) 1.5 M DIBAL-H, toluene, −78 °C (76%).

In his original paper, Garner first protected the amino group with Boc anhydride in dioxane, then esterified the carboxylic acid with iodomethane under basic conditions in dimethylformamide (DMF) and finally formed the dimethyloxazolidine ring of **4** with catalytic *p*-toluenesulfonic acid and 2,2-dimethoxypropane (DMP) in refluxing benzene (Scheme 1) [24–25]. Reduction of the methyl ester **4** to aldehyde **1** was performed with DIBAL-H (175 mol %) at −78 °C. Garner later reported that they had detected some epimerization of the chiral center (5–7% loss of ee down to 93–95% ee) [26]. Another drawback of this route is the use of the toxic and carcinogenic iodomethane in the esterification reaction.

**Scheme 2:**
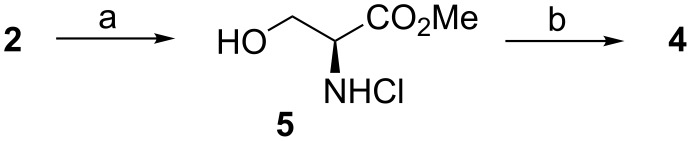
**(**a) AcCl, MeOH, 0 °C → reflux (99%); (b) i) (Boc)_2_O, Et_3_N, THF, 0 °C → rt → 50 °C (89%); ii) Me_2_C(OMe)_2_, BF_3_·Et_2_O, acetone, rt (91%).

Since the first synthesis of aldehyde (*S*)**-1** by Garner there have been many modifications and improvements to the synthesis of the enantiomers ((*R*)**-1** and (*S*)**-1**). Modifications to the original synthesis have focused either on the reaction sequence (esterification first and then Boc protection) or on the reduction to aldehyde. Other groups have tried to improve the synthesis of the aldehyde. McKillop et al. found that the esterification reaction is performed best first with 235 mol % of HCl (formed in situ from the reaction of AcCl with MeOH) in MeOH [27]. The serine methyl ester hydrochloride salt was then protected with Boc anhydride. The *N*,*O*-acetal was formed using BF_3_·Et_2_O and DMP in acetone (Scheme 2). For the reduction they followed Garner’s procedure. This method allows an easy access to the fully protected methyl ester **4**, but the problem of chiral degradation could not be solved (reported rotation **−**89 vs **−**91.7 in [26]).

**Scheme 3:**
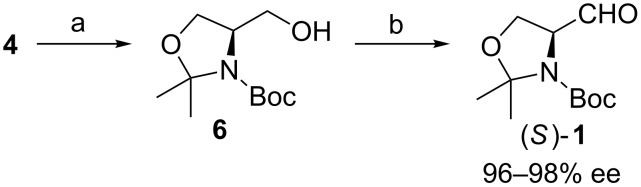
(a) LiAlH_4_, THF, rt (93–96%); (b) (COCl)_2_, DMSO, iPr_2_NEt, CH_2_Cl_2_, −78 °C → −55 °C (99%).

Dondoni adopted McKillop’s procedure for the preparation of **4** [28]. In order to reduce the loss of enantiopurity, they decided to follow Roush’s protocol [29] for the preparation of the aldehyde, i.e. reduction of **4** to alcohol **6** and oxidation of this alcohol to (*S*)**-1**. Moffatt–Swern oxidation provided the final aldehyde (*S*)**-1** from the primary alcohol **6**. In the standard Swern procedure the transformation of the activated alcohol intermediate to the final carbonyl compound (i.e. cleaving off the proton) is done by the addition of Et_3_N at cold temperatures (**–**78 to **–**60 °C). According to Roush, the use of triethylamine for this transformation led to partial racemization of **1** (85% ee). Dondoni noticed that changing the base to the bulkier base *N*,*N*-diisopropylethylamine (Hünig’s base, DIPEA) inhibits the epimerization (Scheme 3) [28]. With DIPEA as the base they could isolate the aldehyde (*S*)**-1** with an enantiopurity of 96–98% ee. The drawback of this route is an additional reaction step, the “overreduction” of the ester **4** to alcohol **6** and the necessary re-oxidation to aldehyde **1**.

As a conclusion of the above results we see that reversing the order of the two first reaction steps has a significant effect on the overall yield (from Garner’s 86% to Dondoni’s 94–98%). Less racemization can be observed with the reduction–oxidation sequence. Of course, other methods for the reduction can be used. The ester can be reduced to alcohol **6** (e.g., with NaBH_4_/LiCl) and then oxidized to **1** with non-basic methods (e.g., IBX/DMP [30] or TEMPO/NaOCl [31] to name a few), which will not epimerize the α-center.

**Scheme 4:**
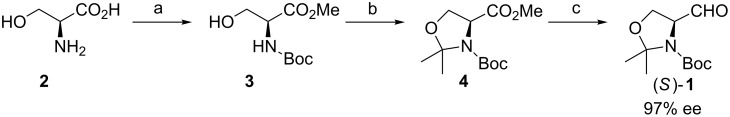
The Koskinen procedure for the preparation of Garner’s aldehyde. (a) i) AcCl, MeOH, 0 °C → 50 °C (99%); ii) (Boc)_2_O, Et_3_N, CH_2_Cl_2_, 0 °C → rt (95–99%); (b) Me_2_C(OMe)_2_, BF_3_·Et_2_O, CH_2_Cl_2_, rt (86%, after high vacuum distillation); (c) DIBAL-H, toluene, −84 °C (EtOAc/N_2_ bath) (82–84%, after high vacuum distillation).

For our synthesis of **1**, we adopted a slightly modified sequence [32]. L-Serine (**2**) is first esterified under traditional Fischer conditions (Scheme 4). The hydrochloride **5** is *N*-protected using (Boc)_2_O. The acetonide is then introduced under mild Lewis acidic conditions to give the desired fully protected serine ester **4**. This is reduced to the aldehyde **1** with DIBAL-H, while keeping the reaction temperature below **−**75 °C. This allowed us to isolate the aldehyde (*S*)**-1** in 97% ee. This reaction sequence was performed in more than 1.0 mol scale starting from L-serine and the reduction to Garner’s aldehyde was performed on a 0.5 mol scale. DIBAL-H is an efficient reducing agent, but at times causes problems during work-up, especially in larger scale reactions. We, among others, have observed the gelatinous aluminium salts after the addition of an aqueous solution. Due to the formation of insoluble gel-like aluminium salts, the extraction procedure gets more challenging and sometimes a small portion of the substrate remains with the aluminium salts. Another drawback of DIBAL-H is the overreduction to alcohol **6**. When only small amounts of the overreduced alcohol are present, one high vacuum distillation is enough to purify the crude aldehyde. When present in amounts greater than 10%, two high vacuum distillations are required. Pure aldehyde **1** crystallizes in the freezer and forms semi-transparent white crystals.

Our procedure provides Garner’s aldehyde (*S*)**-1** in a 66–71% overall yield. The original Garner procedure provides (*S*)**-1** in 46–58% and Dondoni’s in 75–85% yield. Dondoni did neither purify his intermediates (from **2** to (*S*)**-1**) nor the final product. They just reported that all of the products are >95% pure based on NMR analysis.

**Scheme 5:**
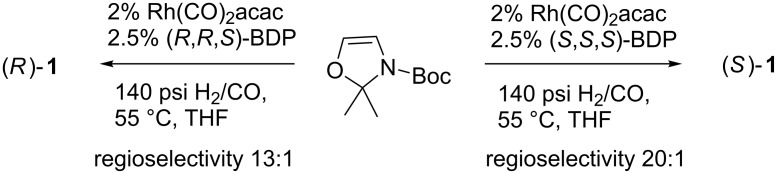
Burke’s synthesis of Garner’s aldehyde. BDP - bis(diazaphospholane).

A very recent approach to the synthesis of Garner’s aldehyde was published by Burke and Clemens [33]. They reported that **1** could be synthesized by asymmetric formylation reaction from Funk’s achiral alkene (Scheme 5) [34]. The formylation reaction provided (*R*)**-1** and (*S*)**-1** in acceptable yields (71% and 70%, respectively) and excellent enantioselectivities (94% ee and 97% ee*,* respectively). However, this synthesis suffers from many drawbacks. Firstly, the synthesis of the Funk’s alkene commences from DL-serine and is a multistep sequence requiring Pb(OAc)_4_. DL-Serine costs 524 €/kg (Aldrich, June 2013 price) compared to L-serine’s 818 €/kg (Aldrich, June 2013). Secondly, both the formylation catalyst and bis(diazaphospholane) ligand are expensive and have to be used in large amounts. Thirdly, the formylation reaction was performed only in a 5 mmol scale and was not optimized for large scale synthesis.

### Asymmetric induction with Garner’s aldehyde

**Figure 3:**
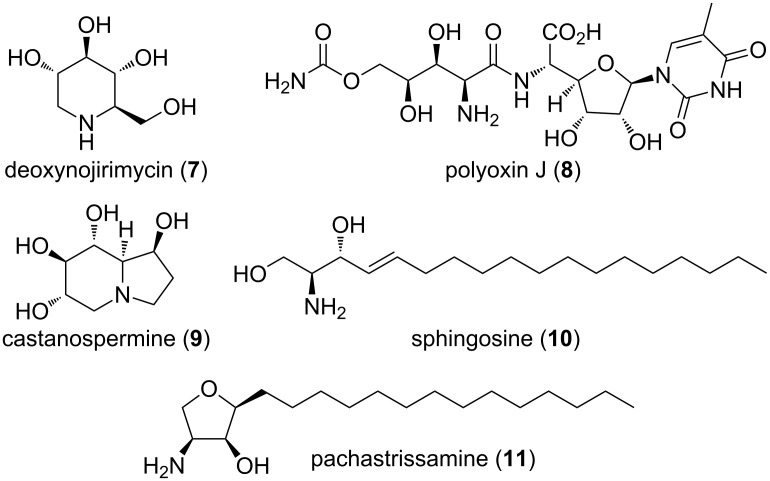
Structures of some iminosugars (**7**, **9**), peptide antibiotics (**8**) and sphingosine (**10**) and pachastrissamine (**11**).

Nucleophilic addition to Garner’s aldehyde gives an easy access to 2-amino-1,3-dihydroxypropyl substructures. This structural motif can be found in many natural products, such as iminosugars (**7** and **9**), peptide antibiotics (**8**), sphingosines and their derivatives (**10** and **11**, Figure 3). These naturally occurring polyhydroxylated compounds have attracted increasing interest from synthetic chemists, because they are frequently found to be potent inhibitors of many carbohydrate-processing enzymes involved in important biological systems. These unique molecules have tremendous potential as therapeutic agents in a wide range of diseases such as metabolic diseases (lysosomal storage disorders, diabetes), viral infections, tumour metastasis, and neurodegenerative disorders.

**Scheme 6:**
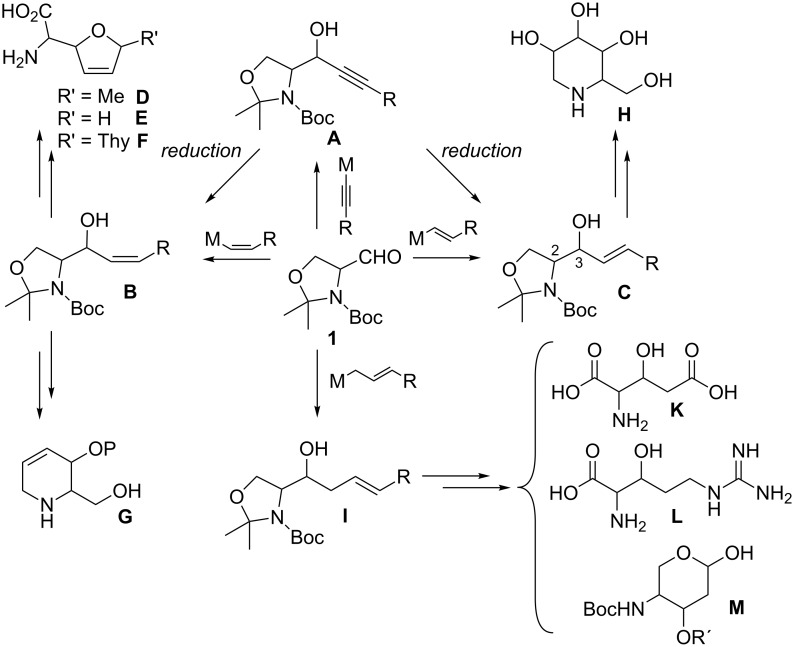
Use of Garner’s aldehyde **1** in multistep synthesis.

Through the addition of a nucleophile to the aldehyde (*S*)**/**(*R*)**-1**, a new C–C bond is formed, hence allowing carbon chain elongation and further functionalization. Nucleophilic addition of an alkyne to **1** gives access to propargylic alcohols of the structure **A** (Scheme 6). This alcohol can be selectively reduced either to *cis*- (**B**) or *trans*-allylic alcohol **C**. *cis*-Selective reduction of **A** can be achieved with Lindlar’s catalyst with H_2_ under atmospheric pressure. The thermodynamic *trans*-allylic alcohol **C** arises from the reaction of **A** with Red-Al. Both isomers **B** and **C** can also be directly accessed from **1** with the corresponding *cis*- and *trans*-vinyl nucleophiles. Allylic alcohol **B** can be used as an intermediate in the synthesis of various natural products or intermediates thereof. The *cis*-double bond allows cyclizations to five- or six-membered rings. Five-membered dihydrofuran rings lead to the synthesis of furanomycin **D** [35–36], norfuranomycin **E** [37], and the polyoxin family **F** [38]. The six-membered tetrahydropyridine **G** synthesized via this route can be used as an intermediate in the synthesis of iminosugars, e.g., of the deoxynojirimycin family **H** [39–43]. The *trans*-allylic alcohol **C** contains already the functional groups of sphingosines and depending on the stereochemistry at C_2_ and C_3_, the synthesis of all four isomers can be achieved [44–47]. Isomer **C** leads also to the synthesis of deoxynojirimycin family **H** [48]. Addition of an allylic nucleophile provides access to homoallylic alcohols **I**. These can be derivatized to unnatural amino acids, such as **K** [49] and **L** [50] or to aminosugar derivatives like **M** [29,51].

Additions of various nucleophiles to **1** have been summarized by Bols and co-workers in 2001 [52]. We have recently reviewed the literature on the synthesis of 1,2-vicinal amino alcohols [53]. Use of Garner’s aldehyde for the synthesis of non-natural amino acids through ethynylglycine has been reviewed [54]. In the following section, significant findings in the use of **1** as an electrophile and chiral intermediate will be discussed.

### Addition of organometallic reagents to Garner’s aldehyde

**Scheme 7:**
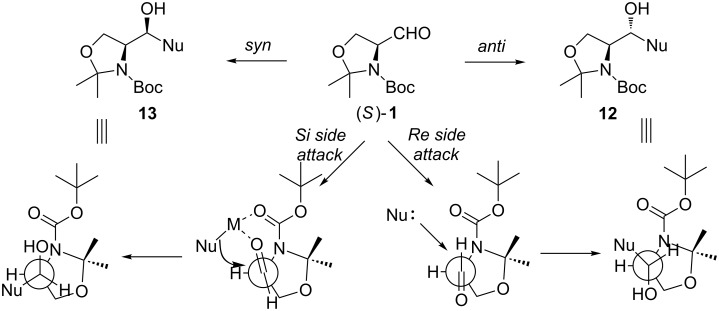
Explanation of the *anti*- and *syn*-selectivity in the nucleophilic addition reaction.

The addition of a nucleophile to Garner’s aldehyde provides a facile access to 2-amino-1,3-dihydroxypropyl substructures. Through the addition of a carbon nucleophile also a new stereocenter is formed. Depending on the stereofacial selectivity, one can access either the *anti*-isomer **12** or *syn*-isomer **13** as the major product (Scheme 7). The high *anti*-selectivity can be rationalized with the attack of the nucleophile from the sterically least hindered side (*re*-side attack). The Felkin–Anh non-chelation transition state model explains this selectivity [55]. The nucleophile attacks not only from the least hindered side (substituent effects), but also from the side where the low-lying σ*C–N orbital is aligned parallel with the π- and π*-orbital of the carbonyl group, allowing delocalization of electron density from the reaction center toward nitrogen. In cases where *syn*-selectivity is observed, the Cram’s chelation control model provides an explanation [56–57]. Chelating metal coordinates between the two carbonyls (the aldehyde and the carbamate), thus forcing the nucleophile to attack from the *si*-side and affecting the selectivity with opposite stereocontrol [58].

**Scheme 8:**
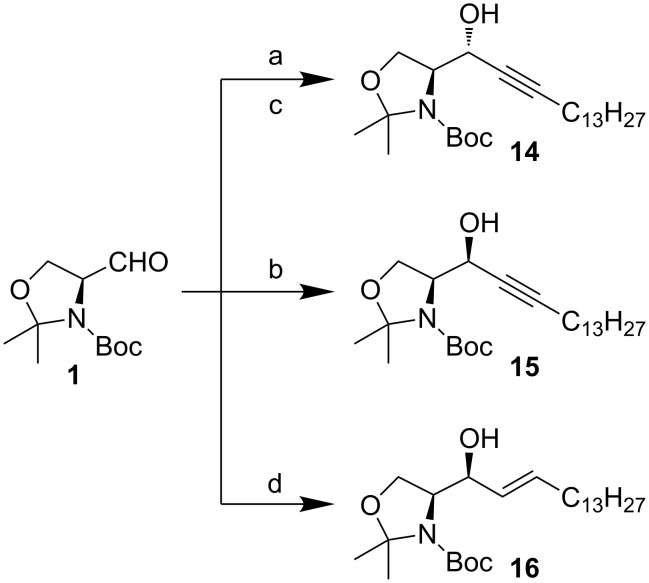
Herold’s method: (a) Lithium 1-pentadecyne, HMPT, THF, −78 °C (71%); (b) Lithium 1-pentadecyne, ZnBr_2_, Et_2_O, −78 °C → rt (87%). Garner’s method: (c) Lithium 1-pentadecyne, THF, −23 °C (83%); (d) 1-Pentadecyne, DIBAL-H, hexanes/toluene, −78 °C (>80%).

In 1988, in pioneering work independently done by Herold [59] and Garner [44] investigated the use of chiral aminoaldehydes as intermediates for the synthesis of nitrogen containing natural products. Both groups realized that the nucleophilic addition of a lithiated alkynyl group to **1** in THF was selective, favouring the *anti*-adduct **14** (Scheme 8). Herold also noticed that the addition of hexamethylphosphorous triamide (HMPT) increased the selectivity from 8:1 (Garner) to >20:1 (*anti*/*syn*). HMPT co-ordinates to the Li-cation, thus breaking the lithium clusters. This increases the nucleophilicity of the alkyne and favours the kinetic *anti*-adduct **14**. Through the use of chelating metals (ZnBr_2_ in Et_2_O [59]) Herold noticed a reversal in selectivity favouring the *syn*-adduct **15** (1:20 *anti*/*syn*). Garner used a slightly different method. He formed the nucleophile reductively from pentadecyne with iBu_2_AlH in THF [44]. This vinylalane provided the *syn*-adduct **16** in modest stereoselectivity (1:2 *anti*/*syn*).

**Scheme 9:**
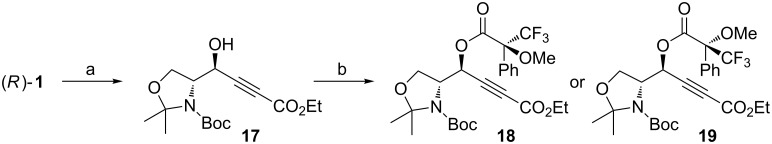
(a) Ethyl lithiumpropiolate, HMPT, THF, −78 °C; (b) (*S*)- or (*R*)-MTPA, DCC, DMAP, THF, rt (**18**, 81%) or (**19**, 87%).

For the addition reaction to be feasible for asymmetric synthesis, the configurational integrity during this step is important. Garner and many others have demonstrated that there is practically no epimerization of the α-carbon center of **1** during the addition reaction [38]. While working on the synthesis of thymine polyoxin C, Garner coupled the lithium salt of ethyl propiolate with (*R*)**-1** (Scheme 9) in HMPT/THF at −78 °C. The reaction was highly *anti*-selective (13:1 *anti*/*syn*) giving adduct **17** in a good yield (75%). The propargylic alcohol **17** was converted to the corresponding Mosher esters [60] **18** and **19**. A careful NMR analysis indicated that there was less than 2% cross-contamination.

**Scheme 10:**
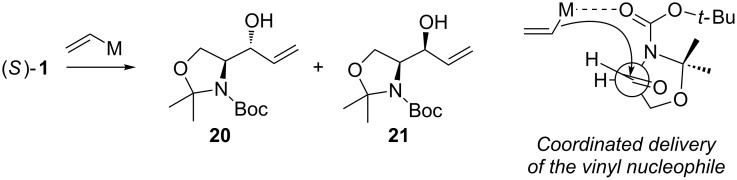
Coleman’s selectivity studies and their transition state model for the co-ordinated delivery of the vinyl nucleophile.

Since the first results of stereoselective additions by Herold and Garner, much attention has been paid on the factors influencing the stereoselectivity. Coleman and Carpenter studied the nucleophilic addition of vinyl organometallic reagents to (*S*)**-1** (Scheme 10) [61]. They noticed that the addition of vinyllithium in THF provided the *anti*-adduct **20** in a moderate 5:1 *anti*/*syn*-selectivity. By changing the metal to magnesium (vinylMgBr) the selectivity slightly dropped to 3:1 (*anti*/*syn*) still favouring adduct **20**. Addition of a Lewis acid (TiCl_4_) did not affect the diastereoselectivity with vinyllithium species. The addition of vinyllithium to (*S*)**-1** in the presence of 100 mol % of TiCl_4_ in THF gave a 5:1 (*anti*/*syn*) mixture of adducts **20** and **21**. Tetrahydrofuran coordinates quite strongly to Lewis acids, which increases the electron density at the metal atom. This lowers the metal atom’s ability to coordinate to other Lewis bases, such as the carbonyl group. By changing the solvent to a poorer donor (≈ less Lewis basic), one can alter the electron density brought about by the solvent molecules to the metal atom. With vinyllithium species in the presence of TiCl_4_ in Et_2_O or toluene, the *anti*-selectivity dropped to 3:1 and 2:1, respectively. Best *syn*-selectivities were achieved with vinylZnCl in Et_2_O (1:6 *anti*/*syn*). Coleman also noticed that the addition of excess ZnCl_2_ did not increase the *syn*-selectivity at all. They attributed this to the mono-coordination of the metal to the carbamate instead of the usual “bidentate” chelation control model (as shown in Scheme 10).

**Scheme 11:**
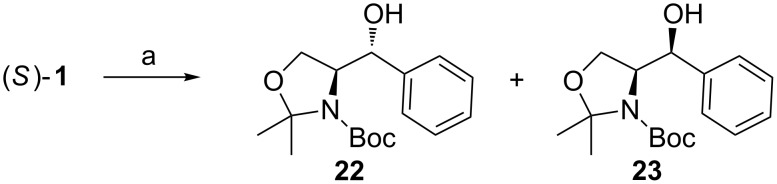
(a) PhMgBr, THF, −78 °C → 0 °C [62] or (a) PhMgBr, Et_2_O, 0 °C [63].

Joullié observed that the more reactive Grignard reagents (e.g., PhMgBr or MeMgBr) give rise to kinetic *anti*-products via the non-chelation pathway [62]. Since these reagents are highly reactive, the reaction takes place before the metal has coordinated to any of the carbonyl groups, thus causing the Felkin–Anh control. When the steric bulk of the nucleophile was increased from PhMgBr to iPrMgBr the selectivity reversed from 5:1 *anti*/*syn* to 1:6 with iPrMgBr. A distinct solvent effect was also observed (Scheme 11). The selectivity obtained by Joullie for the reaction of **1** with PhMgBr was reversed for our system [63]. Joullié obtained a 5:1 (*anti*/*syn*) selectivity of alcohols **22** and **23** in THF compared to the 2:3 ratio observed in Et_2_O. This change in selectivity can be explained by diethyl ether being a less coordinating solvent [64].

**Scheme 12:**
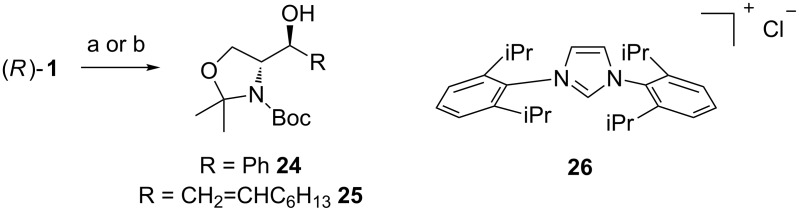
**(**a) cat. RhCl_3_·3H_2_O, cat. **26**, NaOMe, Ph-B(OH)_2_, aq DME, 80 °C (**24**, 71%); (b) cat. RhCl_3_·3H_2_O, cat. **26**, NaOMe, C_6_H_13_CH=CH_2_-B(OH)_2_, aq DME, 55 °C (**25**, 78%).

Fürstner investigated the use of organorhodium nucleophiles with aldehydes (Scheme 12) [65]. They screened catalysts, ligands and bases in order to find the best conditions for the alkylation reaction. They found RhCl_3_·3H_2_O together with imidazolium chloride **26** as the ligand precursor and the base NaOMe to be the catalyst system of choice. NaOMe reacts with the ligand precursor **26** and forms an *N*-heterocyclic carbene. The method was tested also with Garner’s aldehyde and two different boronic acid derived nucleophiles (R = Ph or 1-octenyl). High *anti*-selectivity was observed. With the in situ formed phenyl nucleophile the selectivity was excellent >30:1 (*anti*/*syn*) giving **24** in a good yield (71%), but with the open chain alkene the selectivity eroded to 4.6:1 (*anti*/*syn*) giving **25** in 78% yield. The (*E*)-octenylboronic acid undergoes proto-deborylation at elevated temperatures, so the reaction had to be performed at 55 °C and with slightly higher catalyst loading (5% instead of the usual 3%).

**Scheme 13:**
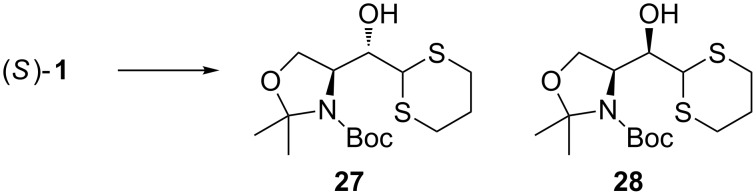
Lithiated dithiane (3 equiv), CuI (0.3 equiv), BF_3_·Et_2_O (6 equiv), THF, −50 °C, 12 h (70%).

Fujisawa studied the addition of lithiated dithiane to (*S*)**-1** (Scheme 13) [66]. Without additives the reaction in THF provided alcohols **27** and **28** in a 7:3 ratio (*anti*/*syn*). Addition of aggregation braking HMPT slightly improved the selectivity (10:3). Almost complete selectivity was achieved when BF_3_·Et_2_O (6 equiv) and CuI (0.3 equiv) were used (>99:1 *anti*/*syn*). They reasoned that the high selectivity arose from the use of a monodentate Lewis acid and the highly dissociated anion derived from the organocopper species.

**Scheme 14:**
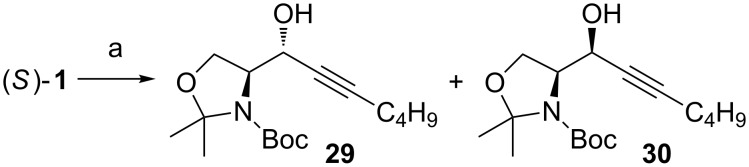
Addition reaction reported by Lam et al. (a) 1-Hexyne, *n*-BuLi, THF, −15 °C or −40 °C.

Recently Lam reported interesting results on the stereoselective addition of a lithiated alkynyl species to (*S*)**-1** (Scheme 14) [67]. According to their results, temperature plays a crucial role on the outcome of the reaction. When the reaction was performed at **–**15 °C (1.36 equiv of alkyne and 1.16 equiv of *n*-BuLi), no *anti*-adduct **29** was detected, instead the *syn*-adduct **30** was obtained as the sole stereoisomer. By lowering the temperature to **−**40 °C and keeping the amount of reagents the same, the selectivity was inverted! Only the *anti*-adduct **29** was isolated. This change in selectivity was explained with two different transition states. At lower temperatures (**−**40 °C) under kinetic control the Felkin–Anh product is predominant. At higher temperatures (**−**15 °C) the nucleophilic addition occurs via a thermodynamically more stable transition state that resembles the chelation control TS. These results are interesting as this is the first time anyone reports a complete inversion of selectivity just by raising the reaction temperature. Decrease in *anti*-selectivity has been evidenced by others, when the reaction temperature has been raised by 40 to 100 °C (e.g., from **−**78 °C to rt), but never a total reverse. If these results are reliable, there has to be a total change in the transition state towards total chelation control and most likely also in the aggregation level of lithiated reagents.

**Scheme 15:**
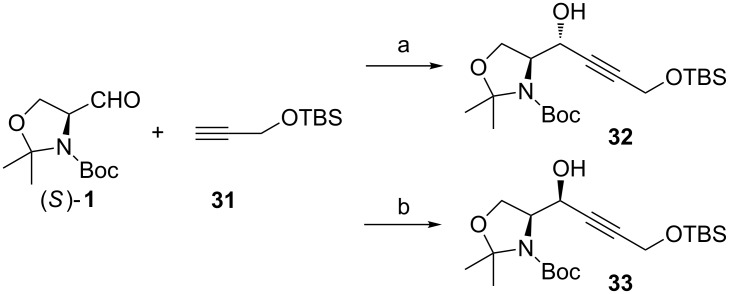
(a) *n*-BuLi, HMPT, toluene, −78 °C → rt (85%); (b) *n*-BuLi, ZnCl_2_, toluene/Et_2_O, −78 °C → rt (65%).

Jurczak has investigated the effect of additives on the selectivity of the nucleophilic addition reaction in toluene [58]. When lithiated **31** was used as the nucleophile, their results were similar to Herold’s [59]. With HMPT as an additive they obtained a selectivity of 20:1 favouring the *anti*-allylic alcohol **32** (Scheme 15). Without additives the selectivity decreased to mere 3:1 (*anti*/*syn*). Under the same conditions but by raising the reaction temperature to rt the selectivity dropped further down to 3:2 (*anti*/*syn*). With chelating metals, such as Mg, Zn and Sn, the stereofacial preference changed from the *re* to the *si* side attack. The use of tin(IV) chloride as the chelating agent gave the highest *syn*-selectivities (>1:20 *anti*:*syn*), but poor yields. Slightly higher yields were achieved with ZnCl_2_ giving the *syn*-adduct **33** in 65% yield, but with lower selectivities (1:10).

**Scheme 16:**
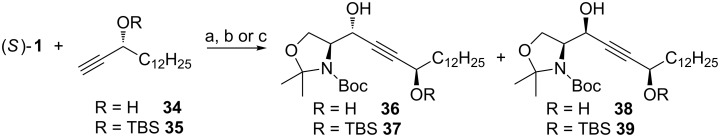
(a) *n-*BuLi, **34**, THF, −40 °C [69]; (b) *n-*BuLi, **35**, THF, −78 °C → rt (80%) [70]; (c) *n*-BuLi, **35**, HMPT, THF, −78 °C (87%) [71].

The additions of propargylic alcohols **34** and **35** were also selective (Scheme 16). Mori performed the addition using unprotected alcohol **34** as nucleophile [68]. The selectivity at **−**40 °C was 4.2:1 favouring the *anti*-adduct **36**. Bittman performed similar addition with **32** as the nucleophile at **−**78 °C [69]. The selectivity was 7.9:1 favouring the *anti*-adduct **37**. In the same year Yadav did the same addition reaction with HMPT which raised the *anti*-selectivity to >20:1 (*anti*/*syn*) [70].

**Scheme 17:**
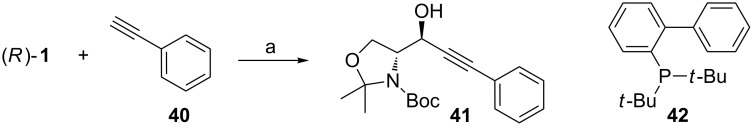
(a) cat. Rh(acac)(CO)_2_, **42**, THF, 40 °C (74%).

Chisholm has been looking for milder catalytic metal-alkyne nucleophiles for carbonyl 1,2-addition reactions, which wouldn’t enolize the labile *α*-protons next to a carbonyl group (Scheme 17) [71]. They found that a Rh(I)-catalyst with a monodentate electron rich phosphine ligand **42** formed a nucleophilic metal-acetylide with terminal alkynes, such as **40**. The phosphine ligand had to be monodentate; bidentate ligands gave a lot lower yields. Their catalyst system worked very efficiently with many carbonyl electrophiles, also with Garner’s aldehyde (*R*)**-1**. The reaction of **1** with **40** gave **41** with high selectivity (>20:1 *anti*/*syn*) in good yield (74%). Unfortunately they do not discuss whether the substrate racemizes under these conditions.

**Scheme 18:**
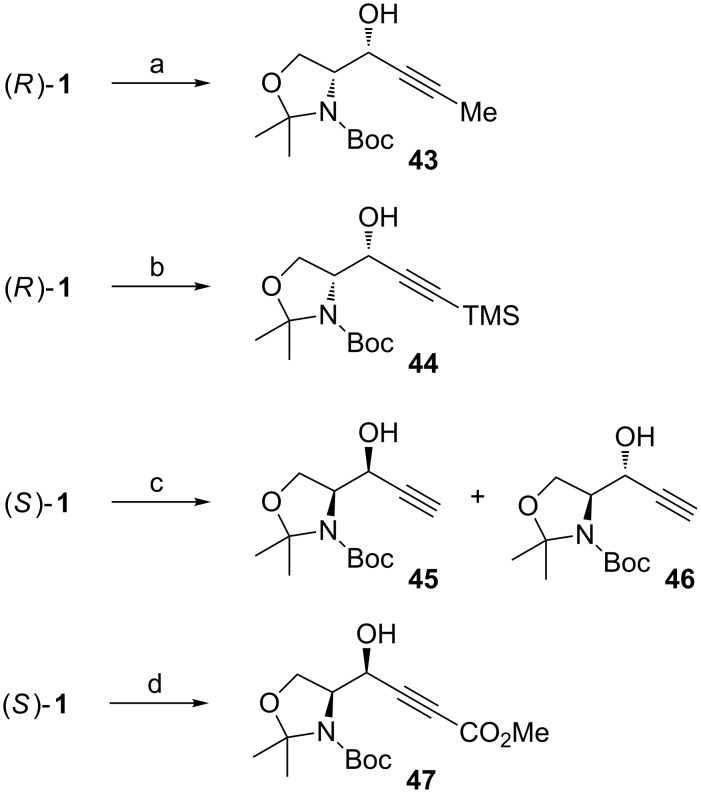
(a) 1-PropynylMgBr, CuI, THF, Me_2_S, −78 °C (95%); (b) Ethynyltrimethylsilane, EtMgBr, CuI, THF, Me_2_S, −78 °C (82%) [72]; (c) EthynylMgCl, ZnBr_2_, toluene, −78 °C [73]; (d) *n*-BuLi, methyl propiolate, Et_2_O, −78 °C → 0 °C, then **(*S*)-1** at −20 °C (62%) [74].

Van der Donk has been interested in the synthesis of dehydro amino acids (Scheme 18) [72]. In two of their examples they used copper-acetylide nucleophiles, which led to high *syn*-selectivities. With propyneCuI the selectivity was 1:16 (*anti*/*syn*) providing the propargylic alcohol **43** in 95% yield. By changing the nucleophile to TMS-ethyneCuI the *syn*-selectivity increased to over 1:20, but providing the *syn*-adduct **44** in lower yield (82%). HPLC analysis showed that no epimerization had occurred. These results support Herold’s seminal work published 20 years earlier [59]. Reginato et al*.* coupled ethyne to Garner’s aldehyde (*S*)**-1** under chelation control [73]. Surprisingly, a 1:1 (*anti*/*syn*) mixture of adducts **45** and **46** was obtained. Hanessian et al*.* have shown that also electron deficient acetylide nucleophiles can be used in the reaction [74]. After carefully studying the reaction conditions and various additives, they found ZnBr_2_ to be the best coordinating agent. The diastereoselectivity was good, favouring the *syn*-adduct **47** (1:12 *anti*/*syn*). The *anti*-selective addition of a lithiopropiolate to (*R*)**-1** was presented by Garner already in 1990 [38].

**Scheme 19:**
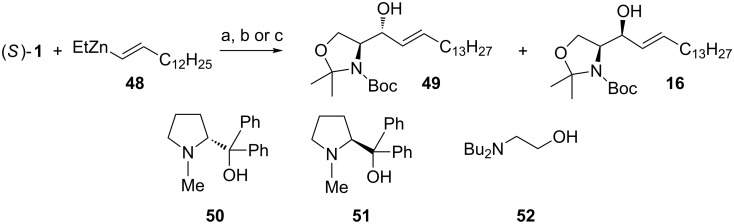
(a) cat. **50**, toluene, 0 °C (52%); (b) cat. **51**, toluene, 0 °C (51%); (c) cat. **52**, toluene, 0 °C (50%).

Soai et al*.* have examined the use of vinylzinc nucleophiles as alkenylating agents in the synthesis of D-*erythro*-sphingosine (Scheme 19) [45]. Treatment of (*S*)**-1** with pentadecenyl(ethyl)zinc (**48**) in the presence of catalytic (*R*)-diphenyl(1-methylpyrrolidin-2-yl)methanol (**50**, (*R*)-DPMPM) [75] in toluene at 0 °C gave adducts **49** and **16** in a 4:1 ratio (*anti*/*syn*). By changing the chiral ligand to (*S*)-DPMPM **51** the selectivity dropped to 2:1 (*anti*/*syn*). When the addition reaction was performed in the presence of achiral *N*,*N*-dibutylaminoethanol **52** they obtained the highest selectivities (7.3:1 *anti*/*syn*). Despite performing the reaction seemingly under chelation control, the selectivity follows the Felkin–Anh transition state model. This selectivity arises from the use of metal coordinating *N*,*O*-ligands. These ligands affect both the reactivity of the metalated nucleophiles and also the metal’s capability of coordination.

**Scheme 20:**
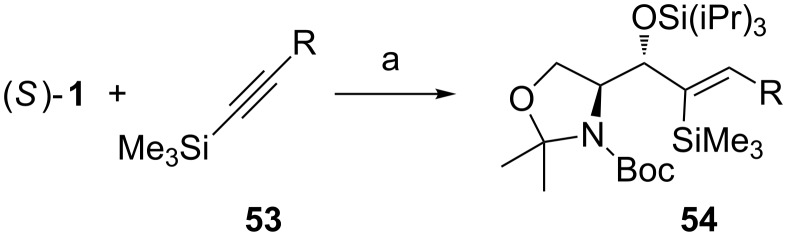
(a) (iPr)_3_SiH, cat. Ni(COD)_2_, dimesityleneimidazolium·HCl, *t*-BuOK, THF, rt.

Montgomery studied the nickel-catalyzed reductive additions of α-aminoaldehydes with silylalkynes (Scheme 20) [76]. The reduction of TMS-alkyne **53** was performed with a trialkylsilane and a Ni(COD)_2_ catalyst ligated with an in situ formed N-heterocyclic carbene. In all cases studied, they found both the *anti*/*syn*- and the *Z*/*E*-selectivity to be high (>20:1), but the chemical yield was varying. Highest yields (78–80%) were achieved with short alkyl chains (R = Me) or when R = phenyl. When the alkyl chain was lengthened (R = C_13_H_27_), while aiming for the synthesis of D-*erythro*-sphingosine, the yield drastically dropped to 18%. By changing the reaction conditions (2 equiv of (*S*)**-1** with 1% water in THF) the yield increased to acceptable levels (65%) while the selectivity remained practically the same. They also tested the reaction with other serinal derivatives, but the best results were achieved with (*S*)**-1**.

**Scheme 21:**
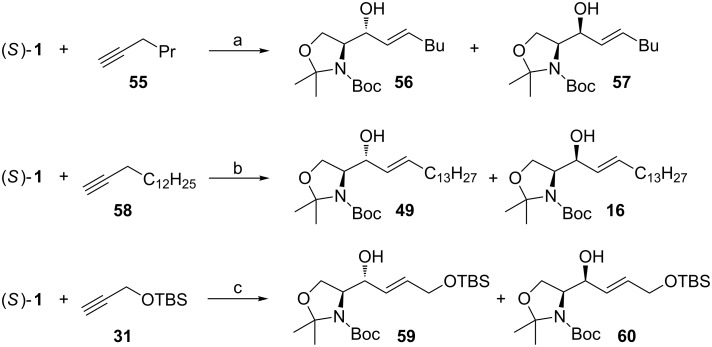
(a) Cp_2_Zr(H)Cl, cat. AgAsF_6_, CH_2_Cl_2_, rt; (b) Cp_2_Zr(H)Cl, 1-pentadecyne, cat. ZnBr_2_ in THF for *anti*-selective or ZnEt_2_ in CH_2_Cl_2_ for *syn*-selective reaction. (c) Cp_2_Zr(H)Cl, Et_2_Zn, in CH_2_Cl_2_, for *syn*-selective reaction or in THF for *anti*-selective reaction.

Alkynes can be converted to (*E*)-vinyl nucleophiles through hydrozirconation. In view of the relatively low electronegativity (1.2–1.4) of Zr, which is roughly comparable with that of Mg and somewhat lower than that of Al, the low reactivity of organylzirconocene chlorides towards carbonyl compounds is puzzling. It is likely that the presence of two sterically demanding cyclopentadienyl (Cp) groups is at least partially responsible for their low reactivity. Suzuki noticed that Lewis acids promote C–C-bond forming reactions of organylzirconocene nucleophiles (Scheme 21) [77]. They used AgAsF_6_ as the Lewis acid promoter and found that also Garner’s aldehyde reacts under these conditions with 1-hexenylzirconocene. The reaction gave a good yield of the addition products **56** and **57** (70% combined yield), but no diastereoselectivity was observed. Peter Wipf has done pioneering work on the hydrozirconation–transmetallation sequence [78–79]. Murakami used this information when investigating the reaction of transmetallated 1-(*E*)-pentadecenylzirconocene chloride with (*S*)**-1** [46–47]. When the reaction was performed in THF at 0 °C and 50 mol % of ZnBr_2_ was added, the reaction gave the *anti*-adduct **49** as the major diastereomer (12:1 *anti*/*syn*). When the amount of ZnBr_2_ was lowered to half (25 mol %) the selectivity rose to 20:1 (*anti*/*syn*). The selectivity was reversed to 1:15 (*anti*/*syn*) favouring the *syn*-adduct **16**, when the solvent was changed to less coordinating CH_2_Cl_2_ and the 1-(*E*)-pentadecenylzirconocene chloride was transmetallated with Et_2_Zn prior to the addition of (*S*)**-1**. Interestingly, when the addition was performed in THF in the presence of the transmetallated 1-(*E*)-pentadecenyl(ethyl)zinc, the selectivity was inverted, and the *anti*-adduct **49** was favoured (12:1 *anti*/*syn*).

**Scheme 22:**
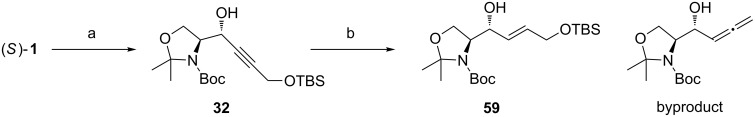
(a) i) **31**, *n*-BuLi, THF, −78 °C; ii) (*S*)-**1**, THF, −78 °C; (b) Red-Al, THF, 0 °C.

We have also been interested in the use of hydrozirconation–transmetallation process while working on the synthesis of galactonojirimycin (Scheme 21) [48]. We used the TBS-protected propargyl alcohol **31** as the nucleophile, which had been investigated by Negishi for the hydrozirconation–transmetallation process [80]. Our findings were in agreement with the results of Murakami. High *syn*-selectivity (>1:20 *anti*/*syn*) was achieved in CH_2_Cl_2_ with Et_2_Zn as the transmetallating agent. The reaction could also be performed in toluene, but the hydrozirconation had to be done in CH_2_Cl_2_ due to the low solubility of the Schwartz’s reagent in toluene. After formation of the hydrozirconated species, the solvent could be changed to toluene. This reaction gave a slightly lower yield in toluene, but identical selectivity favouring adduct **60**. By changing the solvent to THF the stereochemical outcome was reversed. The *anti*-adduct **59** could be isolated in a >20:1 *anti*/*syn* ratio. Unfortunately, the chemical yield was substantially lower (20%) and many byproducts were observed. Most importantly, these reaction conditions did not affect the chiral integrity of (*S*)**-1**. In a recent synthesis of (−)-1-deoxyaltronojirimycin we tried to synthesize the *anti*-adduct **59** [43]. Poor yield for vinylic addition forced us to look for alternative methods (Scheme 22). Despite the literature precedence of *anti*-selective reactions, we were unable to selectively synthesize this adduct in a good yield. We then turned to the lithiated nucleophile **31**. In THF at −78 °C high diastereoselectivity (15:1 *anti*/*syn*) was obtained. Unfortunately, direct reduction of **32** to **59** produced allene as a byproduct, which forced us to modify the synthetic route.

**Scheme 23:**
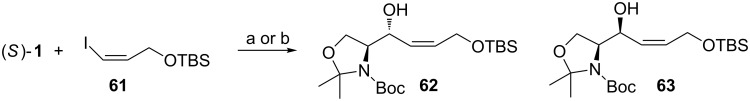
(a) **61**, *n*-BuLi, DMPU, toluene, −78 °C, then (*S*)**-1**, toluene, −95 °C (57%); (b) **61**, *n*-BuLi, ZnCl_2_, toluene, −78 °C, then (*S*)**-1**, toluene, −95 °C (72%).

We have also successfully used (*Z*)-vinyl nucleophiles created from the vinyl iodide **61** in the stereoselective synthesis of pachastrissamine (**11**) [81–82] and norfuranomycin [37] (Scheme 23). After halogen–metal exchange with *n*-BuLi, the newly formed nucleophile reacts with (*S*)**-1**. When HMPT or dimethylpropyleneurea (DMPU) were used, high *anti*-selectivities were achieved (up to 17:1 *anti*/*syn*). Without additives the reaction gave a 4:1 *anti*/*syn* mixture of allylic alcohols **62** and **63**. Chelating metals gave rise to the *syn*-adduct **63**. When we used ZnCl_2_ dissolved in Et_2_O as the chelating agent, the *syn*-selectivity rose to about 1:6 favouring adduct **63**. These results are in agreement with the findings of Jurczak [68].

The alkylation results are summarized in Table 1, and overall one can conclude that Garner’s aldehyde **1** is a highly versatile intermediate for organic synthesis. The selectivity of the 1,2-asymmetric induction can be controlled, either by choice of the nucleophilic reagent, chelating or aggregates breaking additives, solvent and sometimes also by the reaction temperature. Less coordinating metals and more reactive nucleophiles tend to give Felkin–Anh products (Table 1, entries 2, 5, 6, 12–14, 16, 18, 19 and 28). Smaller alkyne nucleophiles seem to give a roughly 8:1 *anti*/*syn*-selectivity. The larger organovinyl reagents give slightly lower *anti*-selectivities of about 3:1 to 5:1. Highest selectivities are reached with transition metals, such as Rh and Ni (Table 1, entries 12, 18 and 19). The *anti*-selectivity can be enhanced either by the addition of aggregation breaking additives, such as HMPT and DMPU (Table 1, entries 1, 4, 10 and 27) or by the use of strongly Lewis basic solvents, like THF (Table 1, entries 2, 5, 13, 14, 16 and 24). Using aggregate breaking additives increases the *anti*-selectivities from 4**–**5:1 to >20:1. The increase of nucleophilicity diminishes the metal cations capability or chances to coordinate to the carbonyl groups. This promotes the attack of the nucleophile from the least hindered *re* side.

**Table 1:** Selectivities and yield for additions of various nucleophiles to Garner’s aldehyde **1**.

Entry	Nucleophile	Additive	Solvent	*T* (°C)	*anti*/*syn*	Yield (%)	Ref.

1	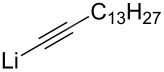	HMPT	THF	−78	>20:1	71	[59]
2	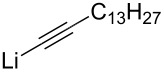	–	THF	−23	8:1	83	[50]
3	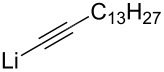	ZnBr_2_	Et_2_O	−78 → rt	1:15	87	[59]
4	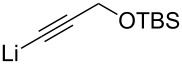	HMPT	toluene	−78 → 0	20:1	85	[58]
5	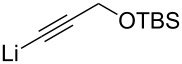	–	THF	−78	15:1	80	[43]
6	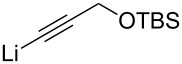	–	toluene	−78 → rt	3:1	80	[58]
7	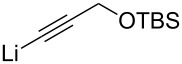	ZnCl_2_	toluene/Et_2_O	−78 → rt	1:10	65	[58]
8	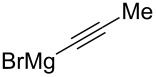	CuI	THF/Me_2_S	−78	1:16	95	[72]
9	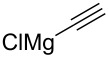	ZnBr_2_	THF/toluene	−78	1:1	–	[73]
10	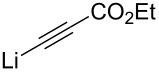	HMPT	THF	−78	13:1	75	[38]
11	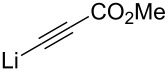	–	Et_2_O	−78 → 0	1:12	62	[74]
12	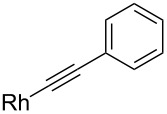	–	THF	40	20:1	74	[71]
13	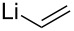	–	THF	−78	5:1	–	[61]
14	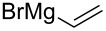	–	THF	−78	3:1	–	[61]
15	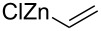	–	Et_2_O	−78 → rt	1:6	85	[61]
16	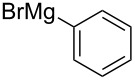	–	THF	−78 → 0	5:1	–	[62]
17	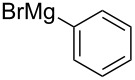	–	Et_2_O	0	2:3	–	[63]
18	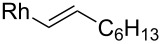	–	aq DME	55	4.6:1	78	[65]
19	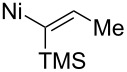	–	THF	rt	>20:1	78	[76]
20	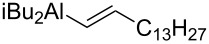	–	THF	−78	1:2	>80	[50]
21	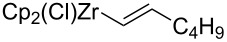	AgAsF_6_	CH_2_Cl_2_	rt	1:1	70	[78]
22	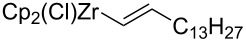	ZnBr_2_	THF	0 → rt	20:1	70	[46]
23	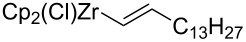	Et_2_Zn	CH_2_Cl_2_	−30 → 0	1:15	84	[46]
24	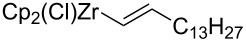	Et_2_Zn	THF	−20 → rt	12:1	67	[46]
25	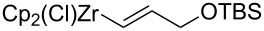	Et_2_Zn	CH_2_Cl_2_	−40 → 0	>1:20	78	[48]
26	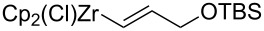	Et_2_Zn	THF	−40 → 0	>20:1	20	[48]
27	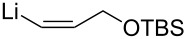	DMPU	toluene	−95	17:1	57	[81–82]
28	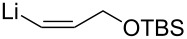	–	toluene	−95	4:1	63	[81–82]
29	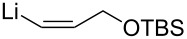	ZnCl_2_	toluene/Et_2_O	−95	1:6	72	[81–82]
30	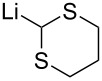	CuI (cat.) BF_3_·Et_2_O	THF	−50	>99:1	70	[66]

Chelation control can also be achieved by the proper choice of solvents. Even changing from THF to Et_2_O is often enough to inverse the selectivity (Table 1, entries 10, 11, 16 and 17). Less coordinating solvents, such as toluene or CH_2_Cl_2_ can also affect the selectivity. A greater effect on the selectivity can be reached by the addition of Lewis acids. Usually Zn(II)-salts give fair to good *syn*-selectivities (Table 1, entries 3, 7, 23, 25 and 29), if the solvent is non-coordinating (Et_2_O, toluene or CH_2_Cl_2_). Zn(II)-salts do not necessarily give *syn*-selectivities, especially if they are too reactive to chelate to the substrate (Table 1, entry 9). Vinylalanes give varying results, preferring either *anti*- or *syn*-selectivity (Table 1, entry 20).

### Olefination of Garner’s aldehyde

**Scheme 24:**
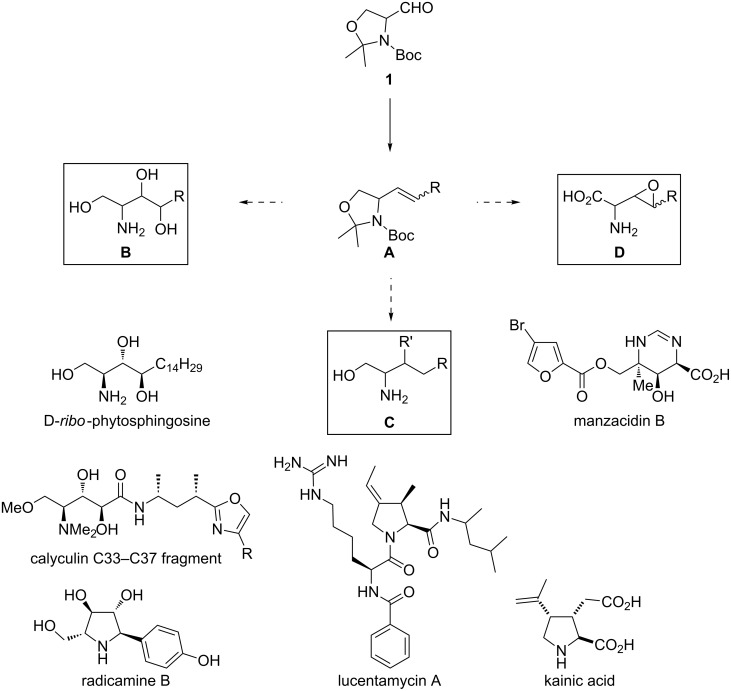
Olefin **A** as an intermediate in natural product synthesis.

Olefination of **1** provides an easy access to chiral 2-aminohomoallylic alcohols **A** (Scheme 24). The intermediate can be derivatized further, thus providing a route for greater molecular diversity. Diastereoselective dihydroxylation of **A** with OsO_4_ leads to triols **B**, which have been utilized in the syntheses of calyculins family [83–84], D-*ribo*-phytosphingosine [85], and radicamine [86–87]. If the R-group in **A** is an ester, diastereoselective Michael addition leads to structures **C** [88–92]. Kainic acid was synthesized using such conjugate addition [93], and a recent synthesis of lucentamycin A was achieved using this strategy [94]. Epoxidation of **A** leads to a highly functional intermediate **C**, which has been used in the synthesis of manzacidin B [95].

Among the plethora of olefination reactions, the Wittig [96–97] and Horner–Wadsworth–Emmons [98–100] reactions are most commonly used for the introduction of a double bond to Garner’s aldehyde **1**. Two things need to be considered before performing the olefination reactions: 1) epimerization of the stereocenter in the aldehyde (i.e. basicity vs nucleophilicity of the olefinating reagents) and 2) *E*/*Z*-selectivity of the reaction.

**Scheme 25:**
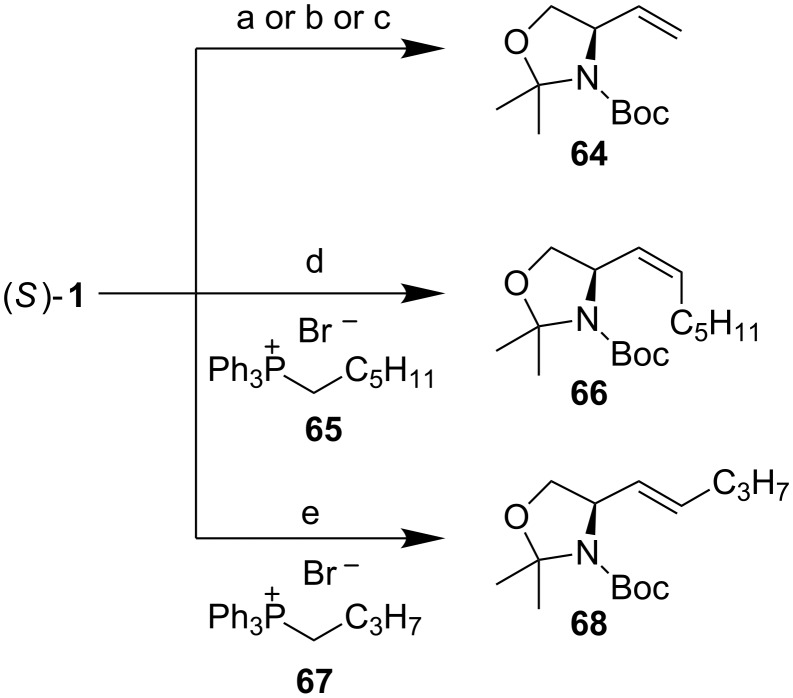
(a) Ph_3_(Me)PBr, KH, benzene (66%, *rac*-**64**) or (b) AlMe_3_, Zn, CH_2_I_2_, THF (76%) [101]; (c) Ph_3_(Me)PBr, *n*-BuLi, THF, −75 °C, then (*S*)-**1** (27%, 69% ee) [102]; (d) **65**, *n*-BuLi, THF, −75 °C, then (*S*)-**1** (78%, 1:13 *E*/*Z*, >95% ee); (e) **67**, KHMDS, THF, −78 °C, then (*S*)-**1**, quenching with MeOH (70%, >10:1 *E*/*Z*) [104].

Moriwake et al. noticed in their synthesis of vinylglycinol **64** that the reaction of methyltriphenylphosphonium bromide with KH as the base provided **62** in fairly good yield, but with complete racemization of the product (Scheme 25) [101]. This racemization could be overcome by performing the olefination with AlMe_3_–Zn–CH_2_I_2_ in benzene under non-basic conditions. While looking for non-racemizing conditions for the same reaction, Beaulieu et al. found that the ylide formed from methyltriphenylphosphonium bromide and *n-*BuLi in THF gave **62** in 69% ee, but in low yield (27%) [102]. With a different unstabilized ylide **65**, the enantiopurity of the Wittig product increased to >95% ee. The erosion of enantiopurity was attributed to the ylide (Ph_3_P=CH_2_) being too basic. It is well known that phosphonium ylides form stable complexes with alkali metals during the dehydrohalogenation of the phosphonium salt [103]. These complexes react with carbonyl compounds differently. Addition reactions of other ylides to **1**, proceeded with little or no racemization of **1**. The *E*/*Z*-ratio of the reaction was 1:13 favouring the *Z*-adduct **66**. In comparison to Beaulieu’s results, McKillop [27] noticed that with KHMDS as the base, there was no epimerization at all! To prepare “salt-free” ylides, it is necessary to remove lithium halide from the reaction solution. On the other hand KHMDS is a convenient base for the preparation of “salt-free” ylides.

High *Z*-selectivity is a typical outcome with unstabilized ylides. Kim has investigated means of reversing the *E*/*Z*-selectivity to favour the *E*-isomer [104]. They noticed that olefination under the usual Wittig conditions provided high *Z*-selectivity (1:15 *E*/*Z*). When the reaction was quenched with MeOH at −78 °C, the *E*/*Z*-ratio was reversed to >10:1 favouring adduct **68** [105].

**Scheme 26:**
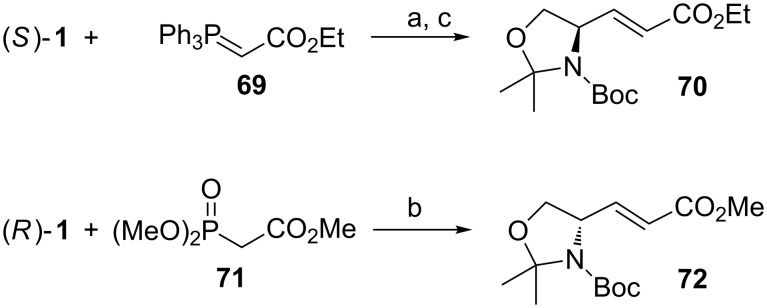
(a) Benzene, rt (82%) [108]; (b) K_2_CO_3_, MeOH (85%) [89]; (c) iPrOH, [Ir(COD)Cl]_2_, PPh_3_, THF, rt (81%) [114].

α,β-Unsaturated esters can be synthesized from stabilized ylides. Reaction of **1** with ylide **69** is *E*-selective. Depending on the solvent, the *E*/*Z*-ratio can vary from 3:1 (in MeOH) [106] to 100:0 (THF [107] or benzene [108]). Since stabilized ylides are less reactive compared to their non-stabilized counterparts, they tend to epimerize the existing chiral center of **1** a lot less, if at all (p*K*_aH_ value of **69** in DMSO is 8.5 compared to the p*K*_a_ value of Ph_3_(Me)PBr, which is 22.5) [109]. Another method to prepare α,β-unsaturated esters is the Horner–Wadsworth–Emmons reaction (HWE). The HWE reaction has many advantages over the Wittig olefination. The phosphonate anions tend to be more nucleophilic (less basic) than the corresponding phosphorous ylides. The byproducts, dialkyl phosphates are water soluble and hence easier to remove from the product compared to, e.g., triphenylphospine oxide. As with the Wittig reaction, the HWE reaction can also promote the epimerization of the α-proton (p*K*_a_ of triethoxyphosphonoacetate in DMSO is 18.6) [109]. We have experienced this tendency especially with α-amino ketophosphonates [110], but there is also evidence that aldehyde **1** can lose some of its chiral integrity in the HWE reaction. For base sensitive substrates the use of metal salts (LiCl or NaI) and an amine base (DBU or DIPEA) has proven to be effective in avoiding epimerization [111]. The use of Ba(OH)_2_ in aq THF has also been advocated to prevent epimerization [112], and recently Myers has reported the superiority of lithium hexafluoroisopropoxide as a mild base for HWE olefinations of epimerizable aldehydes [113]. Jako et al*.* showed that *E*-enoate **72** can be synthesized from Garner’s aldehyde (*R*)**-1** in 95:5 *E*/*Z*-selectivity and practically with no degradation of chiral integrity (Scheme 26) [89]. As an alternative, Lebel and Ladjel used a catalytic amount of [Ir(COD)Cl]_2_ for the in situ preparation of ylide **69** [114]. They obtained a 81% yield in the reaction.

**Scheme 27:**
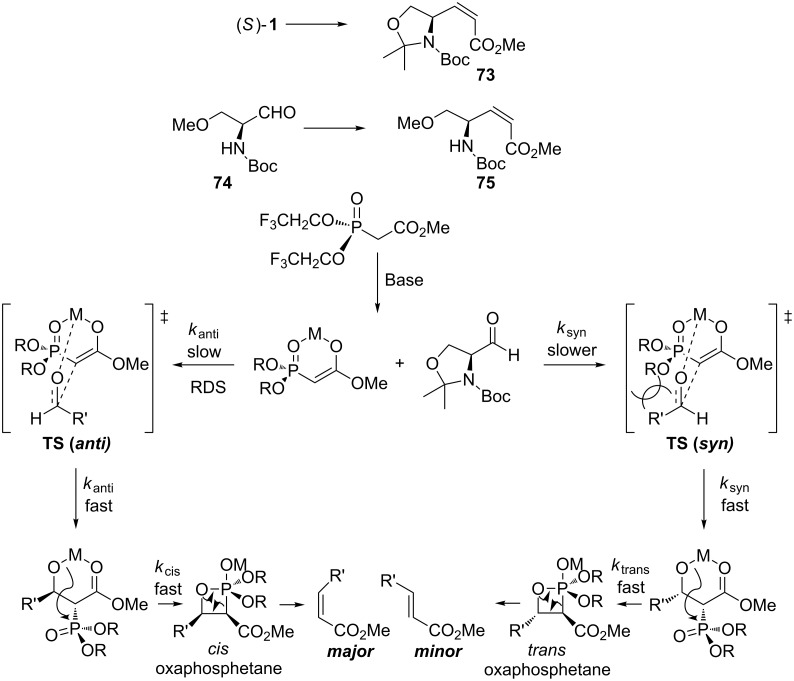
Mechanism of the Still–Gennari modification of the HWE reaction leading to both olefin isomers.

We have been interested in the synthesis of *Z*-enoate **73** [84]. The Still–Gennari modification to the phosphonate makes the synthesis of *Z*-enoates possible [115]. The two electron-withdrawing CF_3_CH_2_O-groups destabilize the *cis*-oxaphosphetane intermediate (Scheme 27) and make the elimination reaction to the kinetic product *Z*-alkene a lot faster. As the elimination step becomes fast, the rate difference in the initial addition step between *k*_anti_ and *k*_syn_ determines the overall *Z*-selectivity. When the reaction was performed with K_2_CO_3_/18-crown-6 as the base in toluene at **−**15 °C, we could isolate only the *Z*-enoate **73** in good yield (90%). HPLC analysis showed that there was no epimerization.

We have recently shown that the open-chain aldehyde **74** reacts with the Still–Gennari phosphonate and provides the *Z*-enoate **75** in good *E*/*Z*-selectivity (1:12) [84]. The slight decrease in the *E*/*Z*-selectivity can be reasoned with the smaller size of the aldehyde **74**. When the aldehyde is coordinating to the phosphonate, the steric hindrance caused by the interaction of the trifluoroethoxy group with the aldehyde R’ in **TS (*syn*)** is smaller compared with aldehyde **1**. This allows some of the aldehyde to react via the *trans*-oxaphosphetane intermediate. Using these reaction conditions no epimerization was observed.

## Conclusion

Garner’s aldehyde has developed into a useful and reliable synthetic intermediate for the synthesis of enantiopure complex natural products, their analogues and other pharmacologically active compounds. Several reaction types have been studied sufficiently so that one has reliable tools to plan a synthesis. Simple addition reactions to the carbonyl group give access to vicinal amino alcohols, important building blocks for many natural products. Another possibility for carbon chain elongation are olefination reactions, which often lead to epimerization of the α-stereocenter. As with the alkylations, strategies to avoid this undesired reaction have been developed. However, despite these important achievements, much more research needs to be done to increase the scopes of these reactions, the overall efficiency, and environmental sustainability.

## References

[1] Vallery-Radot R, Devonshire R L (1960). Life of Pasteur (transl.).

[2] Ramos Tombo G M, Belluš D (1991). Angew Chem, Int Ed Engl.

[3] Lamberth C (2010). Tetrahedron.

[4] Vaenkatesan V, Wegh R T, Teunissen J-P, Lub J, Bastiaansen C W M, Broer D J (2005). Adv Funct Mater.

[5] Zhuang X X, Sun X X, Li L, Li Y C (2011). Adv Mat Res.

[6] Armstrong R W, Beau J M, Cheon S H, Christ W J, Fujioka H, Ham W H, Hawkins L D, Jin H, Kang S H, Kishi Y (1989). J Am Chem Soc.

[7] Armstrong R W, Beau J M, Cheon S H, Christ W J, Fujioka H, Ham W H, Hawkins L D, Jin H, Kang S H, Kishi Y (1989). J Am Chem Soc.

[8] Suh E M, Kishi Y (1994). J Am Chem Soc.

[9] Mangeney P, Andriamialisoa R Z, Langlois N, Langlois Y, Potier P (1979). J Am Chem Soc.

[10] Kutney J P, Choi L S L, Nakano J, Tsukamoto H, McHugh M, Boulet C A (1988). Heterocycles.

[11] Kuehne M E, Matson P A, Bornmann W G (1991). J Org Chem.

[12] Magnus P, Mendoza J S, Stamford A, Ladlow M, Willis P (1992). J Am Chem Soc.

[13] Yokoshima S, Ueda T, Kobayashi S, Sato A, Kuboyama T, Tokuyama H, Fukuyama T (2002). J Am Chem Soc.

[14] Ishikawa H, Colby D A, Seto S, Va P, Tam A, Kakei H, Rayl T J, Hwang I, Boger D L (2009). J Am Chem Soc.

[15] Nicolaou K C, Yang Z, Liu J J, Ueno H, Nantermet P G, Guy R K, Claiborne C F, Renaud J, Couladouros E A, Paulvannan K (1994). Nature.

[16] Holton R A, Somoza C, Kim H B, Liang F, Biediger R J, Boatman P D, Shindo M, Smith C C, Kim S, Nadizadeh H (1994). J Am Chem Soc.

[17] Holton R A, Somoza C, Kim H B, Liang F, Biediger R J, Boatman P D, Shindo M, Smith C C, Kim S, Nadizadeh H (1994). J Am Chem Soc.

[18] Danishefsky S J, Masters J J, Young W B, Link J T, Snyder L B, Magee T V, Jung D K, Isaacs R C A, Bornmann W G, Alaimo C A (1996). J Am Chem Soc.

[19] Wender P A, Badham N F, Conway S P, Floreancig P E, Glass T E, Gränicher C, Houze J B, Jaenichen J, Lee D, Marquess D G (1997). J Am Chem Soc.

[20] Wender P A, Badham N F, Conway S P, Floreancig P E, Glass T E, Houze J B, Krauss N E, Lee D, Marquess D G, McGrane P L (1997). J Am Chem Soc.

[21] Morihira K, Hara R, Kawahara S, Nishimori T, Nakamura N, Kusama H, Kuwajima I (1998). J Am Chem Soc.

[22] Mukaiyama T, Shiina I, Iwadare H, Saitoh M, Nishimura T, Ohkawa N, Sakoh H, Nishimura K, Tani Y-I, Hasegawa M (1999). Chem–Eur J.

[23] Doi T, Fuse S, Miyamoto S, Nakai K, Sasuga D, Takahashi T (2006). Chem–Asian J.

[24] Garner P (1984). Tetrahedron Lett.

[25] Garner P, Park J M (1990). Org Synth.

[26] Garner P, Park J M (1987). J Org Chem.

[27] McKillop A, Taylor R J K, Watson R J, Lewis N (1994). Synthesis.

[28] Dondoni A, Perroni D (2000). Org Synth.

[29] Roush W R, Hunt J A (1995). J Org Chem.

[30] Ocejo M, Vicario J L, Badía D, Carrillo L, Reyes E (2005). Synlett.

[31] Jurczak J, Gryko D, Kobrzycka E, Gruza H, Prokopowicz P (1998). Tetrahedron.

[R32] 32Rauhala, V. Master’s thesis, Department of Chemistry, University of Oulu, **1998**, p. 87.

[33] Clemens A J L, Burke S D (2012). J Org Chem.

[34] Huntley R J, Funk R L (2006). Org Lett.

[35] Chattopadhyay S K, Sarkar K, Karmakar S (2005). Synlett.

[36] Bandyopadhyay A, Pal B K, Chattopadhyay S K (2008). Tetrahedron: Asymmetry.

[37] Passiniemi M, Koskinen A M P (2011). Tetrahedron Lett.

[38] Garner P, Park J M (1990). J Org Chem.

[39] Takahata H, Banba Y, Ouchi H, Nemoto H (2003). Org Lett.

[40] Takahata H, Banba Y, Sasatani M, Nemoto H, Kato A, Adachi I (2004). Tetrahedron.

[41] Guaragna A, D’Errico S, D’Alonzo D, Pedatella S, Palumbo G (2007). Org Lett.

[42] Guaragna A, D’Alonzo D, Paolella C, Palumbo G (2009). Tetrahedron Lett.

[43] Karjalainen O K, Koskinen A M P (2011). Org Biomol Chem.

[44] Garner P, Park J M, Malecki E (1988). J Org Chem.

[45] Soai K, Takahashi K (1994). J Chem Soc, Perkin Trans 1.

[46] Murakami T, Furusawa K (2002). Tetrahedron.

[47] Murakami T, Furusawa K, Tamai T, Yoshikai K, Nishikawa M (2005). Bioorg Med Chem Lett.

[48] Karjalainen O K, Passiniemi M, Koskinen A M P (2010). Org Lett.

[49] Tamborini L, Conti P, Pinto A, Colleoni S, Gobbi M, De Micheli C (2009). Tetrahedron.

[50] Lemke A, Büschleb M, Ducho C (2010). Tetrahedron.

[51] Chisholm J D, Van Vranken D L (2000). J Org Chem.

[52] Liang X, Andersch J, Bols M (2001). J Chem Soc, Perkin Trans 1.

[53] Karjalainen O K, Koskinen A M P (2012). Org Biomol Chem.

[54] Reginato G, Meffre P, Gaggini F (2005). Amino Acids.

[55] Ahn N T (1980). Top Curr Chem.

[56] Cram D J, Abd Elhafez F A (1952). J Am Chem Soc.

[57] Reetz M T, Hüllmann M, Seitz T (1987). Angew Chem, Int Ed Engl.

[58] Gruza H, Kiciak K, Krasiński A, Jurczak J (1997). Tetrahedron: Asymmetry.

[59] Herold P (1988). Helv Chim Acta.

[60] Dale J A, Dull D L, Mosher H S (1969). J Org Chem.

[61] Coleman R S, Carpenter A J (1992). Tetrahedron Lett.

[62] Williams L, Zhang Z, Shao F, Carroll P J, Joullié M M (1995). Tetrahedron.

[63] Koskinen A M P, Hassila H, Myllymäki V T, Rissanen K (1995). Tetrahedron Lett.

[64] Marcus Y (1998). The Properties of Solvents.

[65] Fürstner A, Krause H (2001). Adv Synth Catal.

[66] Shimizu M, Wakioka I, Fujisawa T (1997). Tetrahedron Lett.

[67] Wong L, Tan S S L, Lam Y, Melendez A J (2009). J Med Chem.

[68] Mori K, Masuda Y (2003). Tetrahedron Lett.

[69] Chun J, Byun H-S, Bittman R (2003). J Org Chem.

[70] Yadav J S, Geetha V, Krishnam Raju A, Gnaneshwar D, Chandrasekhar S (2003). Tetrahedron Lett.

[71] Dhondi P K, Carberry P, Choi L B, Chisholm J D (2007). J Org Chem.

[72] Zhang X, van der Donk W A (2007). J Am Chem Soc.

[73] Reginato G, Mordini A, Tenti A, Valacchi M, Broguiere J (2008). Tetrahedron: Asymmetry.

[74] Hanessian S, Yang G, Rondeau J-M, Neumann U, Betschart C, Tinelnot-Blomley M (2006). J Med Chem.

[75] Soai K, Ookawa A, Kaba T, Ogawa K (1987). J Am Chem Soc.

[76] Sa-ei K, Montgomery J (2009). Tetrahedron.

[77] Suzuki K, Hasegawa T, Imai T, Maeta H, Ohba S (1995). Tetrahedron.

[78] Wipf P, Xu W (1994). Tetrahedron Lett.

[79] Wipf P, Jahn H (1996). Tetrahedron.

[80] Xu C, Negishi E (1999). Tetrahedron Lett.

[81] Passiniemi M, Koskinen A M P (2008). Tetrahedron Lett.

[82] Passiniemi M, Koskinen A M P (2011). Org Biomol Chem.

[83] Koskinen A M P, Chen Y (1991). Tetrahedron Lett.

[84] Passiniemi M, Koskinen A M P (2010). Synthesis.

[85] Jeon J, Shin M, Yoo J W, Oh J S, Bae J G, Jung S H, Kim Y G (2007). Tetrahedron Lett.

[86] Ribes C, Falomir E, Carda M, Marco J A (2008). J Org Chem.

[87] Mallesham P, Vijaykumar B V D, Shin D-S, Chandrasekhar S (2011). Tetrahedron Lett.

[88] Jako I, Uiber P, Mann A, Taddei M, Wermuth C G (1990). Tetrahedron Lett.

[89] Yoda H, Shirai T, Katagiri T, Takabe K, Kimata K, Hosoya K (1990). Chem Lett.

[90] Hanessian S, Sumi K (1991). Synthesis.

[91] Hanessian S, Demont E, van Otterlo W A L (2000). Tetrahedron Lett.

[92] Rastogi S K, Kornienko A (2006). Tetrahedron: Asymmetry.

[93] Jako I, Uiber P, Mann A, Wermuth C-G (1991). J Org Chem.

[94] Ranatunga S, Tang C-H A, Hu C-C A, Del Valle J R (2012). J Org Chem.

[95] Sankar K, Rahman H, Das P P, Bhimireddy E, Sridhar B, Mohapatra D K (2012). Org Lett.

[96] Wittig G, Schöllkopf U (1954). Chem Ber.

[97] Wittig G, Haag W (1955). Chem Ber.

[98] Horner L, Hoffmann H, Wippel H G (1958). Chem Ber.

[99] Horner L, Hoffmann H, Wippel H G, Klahre G (1959). Chem Ber.

[100] Wadsworth W S, Emmons W D (1961). J Am Chem Soc.

[101] Moriwake T, Hamano S, Saito S, Torii S (1987). Chem Lett.

[102] Beaulieu P L, Duceppe J-S, Johnson C (1991). J Org Chem.

[103] Kolodiazhnyi O I (1999). Phosphorus ylides – Chemistry and Application in Organic Synthesis.

[104] Oh J S, Kim B H, Kim Y G (2004). Tetrahedron Lett.

[105] Schlosser M, Christmann K F (1966). Angew Chem, Int Ed Engl.

[106] Drew M G B, Harrison R J, Mann J, Tench A J, Young R J (1999). Tetrahedron.

[107] Upadhyay P K, Kumar P (2010). Synthesis.

[108] Dondoni A, Merino P, Perrone D (1993). Tetrahedron.

[109] Bordwell F G (1988). Acc Chem Res.

[110] Pelšs A, Kumpulainen E T T, Koskinen A M P (2009). J Org Chem.

[111] Blanchette M A, Choy W, Davis J T, Essenfeld A P, Masamune S, Roush W R, Sakai T (1984). Tetrahedron Lett.

[112] Paterson I, Yeung K-S, Smaill J B (1993). Synlett.

[113] Blasdel L K, Myers A G (2005). Org Lett.

[114] Lebel H, Ladjel C (2008). Organometallics.

[115] Still W C, Gennari C (1983). Tetrahedron Lett.

